# A new role for inflammasomes: sensing the disturbances in non-alcoholic fatty liver disease

**DOI:** 10.3389/fphys.2013.00156

**Published:** 2013-07-01

**Authors:** Poorvi Desai, Prasanna Tamarapu Parthasarathy, Lakshmi Galam, Richard Lockey, Narasaiah Kolliputi

**Affiliations:** Department of Internal Medicine, Division of Allergy and Immunology, Morsani College of Medicine, University of South FloridaTampa, FL, USA

Non-alcoholic fatty liver disease (NAFLD) is a manifestation of metabolic syndrome, a group of conditions including hypertension, high plasma glucose, excess body fat around waist or abnormal cholesterol levels which will work together to increase the risk of heart disease, diabetes, and stroke (Kim and Younossi, 2008). Although 20% of NAFLD cases progress to non-alcoholic steatohepatitis (NASH), the exact cause of the disease progression is uncertain (Lucy Bird, 2012; Ibrahim et al., 2013). Gut and liver are connected via the portal circulation and through embryogenesis they are clearly linked. Researchers hypothesized that due to this link, imbalances in the gut could influence liver disease. Normal gut flora plays a significant role in keeping our innate immune system intact and when dysbiosis occurs, it may lead to various inflammatory diseases (Wood, 2012). Inflammasomes are recently discovered multiprotein complexes involved in a diverse range of inflammatory pathologies (Kolliputi et al., 2010, 2012). A specific inflammasome sensor consists of nucleoside-triphosphatase domain (NACHT), leucine rich repeat (LRR), and pyrin domain (PYD), which are domains-containing protein 3 (NLRP3) (Lane et al., 2013; Qazi et al., 2013). Toll-like receptors (TLRs) are a different subset of “danger-sensing” receptors that trigger proinflammatory gene expression (Janssens and Beyaert, 2003).

An exciting but different way at looking at the progression of NAFLD to NASH has been conducted by Henao-Mejia et al. (2012). Though the association between intestinal dysbiosis and progression of FLD has been studied in the past, Henao-Mejia et al. now suggest that gut dysbiosis may be the driver of this progression, not just an association. To carry out this study, the researchers looked at the inflammasomes NLRP6 and NLRP3, and a cytokine, Interleukin-18 (IL-18). Since the major aim of this study was to determine the cause of progression of NAFLD in Metabolic Syndrome, mice were fed a methionine-choline-deficient diet (MCDD) to induce FLD. One way that inflammasomes corrected the imbalance was through activating IL-1 and IL-18. Additionally, it was shown that with the absence of inflammasomes, the gut flora was not symbiotic and TLRs 4 and 9 were able to be delivered to the liver. These TLRs made the FLD worse and exacerbated NASH by producing TNF-α, thereby showing a link between the gut-mediating inflammasomes and the progression of NAFLD. The NLRPs seem to work as gate-keepers in keeping TLR's normally. The authors found that mice lacking the NLRP- inflammasomes, or in other words did not have the danger-sensing mechanism, allowed the habitation of two abnormal microbial species in their gut, *Prevotella* and *Porphyromondaceae*. Also, when inflammasome-deficient MCDD mice were cohabited with wild type (WT) mice, the dysbiosis and manifestation of disease were transferred over to the WT mice. Additionally, they found that the exacerbation of FLD in the inflammasome-deficient WT mice did not rely on hematopoietic or hepatocyte inflammasomes, implicating an alternate cell population in controlling intestinal homeostasis. Since intestinal epithelial cells possess inflammasomes, it was concluded that they were the reason for transfer. IL-18 has previously been shown to maintain the integrity of these epithelial cells, and it was shown in this study, consistent with other studies that an impaired IL-18 led to an exacerbation of NAFLD (Elinav et al., 2011).

To investigate the progression of Metabolic Syndrome, Henao-Mejia et al., studied an obese mice model, the leptin-receptor deficient *db/db* mice that developed NAFLD, impaired intestinal barrier function, and glucose intolerance when fed a high fat diet (HFD). They found that the *db/db* mice cohabited with inflammasome-deficient mice had an increase in liver disease progression, including increased hepatocyte injury, steatosis, and inflammation when compared to *db/db* mice alone. Furthermore, the gut flora in HFD mice was involved in worsening metabolic symptoms, which include obesity, glucose intolerance, and insulin resistance. Once again when given antibiotics, the situation reversed itself. Previous studies have shown inflammasomes to be responsible for inflammatory destruction, implying that different inflammasomes carry different functions (Masters, 2013). As mentioned earlier, IL-18 seems crucial in maintaining homeostasis. IL-1, on the other hand, is implicated in developing metabolic disease syndrome and so deemed pro-inflammatory to worsen the FLD. For example, Stienstra et al. have found that in the obese mice, a HFD leads to adipose tissue inflammation by NLRP3 activators like ceramide. The NLRP3-KO mice were resistant to the development of HFD-induced obesity, insulin resistance, and hepatic steatosis in an IL-1 dependent manner (Stienstra et al., 2011; Vandanmagsar et al., 2011). With this apparent difference between IL-1 and IL-18, targeting cytokines and inflammasomes may prove to be of great therapeutic benefit.

This study (Henao-Mejia et al., 2012) paves the way toward exciting new treatment possibilities for FLD progression and exacerbation. By looking at the gut-liver axis it shows that dysbiosis in the gut flora can be the cause for FLD. Dysbiosis came about through the absence of NLRP3 and NLRP6 inflammasomes with the presence of IL-18. Furthermore, gut flora disturbances can occur through a complex interplay between genes and the environment. Maintaining normal gut microbiota might be the key in stopping the harmful effects of metabolic syndrome, namely the progression of inflammatory liver diseases.
